# A model for predicting overall survival in bladder cancer patients with signet ring cell carcinoma: a population-based study

**DOI:** 10.1186/s40001-022-00970-y

**Published:** 2023-02-02

**Authors:** Liang Liu, Chuangui Li, Qiang Wang, Haibo Yuan, Yuanyuan Wang

**Affiliations:** Department of Urology, Prostate & Andrology Key Laboratory of Baoding, Baoding No. 1 Central Hospital, No. 320 Changcheng North Street, Lianchi District, Baoding, 071000 Hebei China

**Keywords:** Signet ring cell carcinoma, Urinary bladder neoplasms, Nomogram, OS, SEER

## Abstract

**Introduction:**

This study is to examine the predictors of survival and to construct a nomogram for predicting the overall survival (OS) of primary bladder signet ring cell carcinoma (SRCC) patients based on the analysis of the Surveillance, Epidemiology, and End Results (SEER) database.

**Methods:**

A total of 219 eligible patients diagnosed with SRCC were analyzed using the 2004–2015 data from SEER database. Univariate and multivariate Cox regression were used to determine independent prognostic factors, followed by development of a nomogram based on the multivariate Cox regression models. The consistency index (C-index), receiver operating characteristic (ROC) curve, and calibration curve were used to validate the prognostic nomogram.

**Results:**

The nomograms indicated appreciable accuracy in predicting the OS, with C-index of 0.771 and 0.715, respectively. The area under the curve (AUC) of the nomogram was 0.713 for 1 year, 0.742 for 3 years, and 0.776 for 5 years in the training set, while was 0.730 for 1 year, 0.727 for 3 years, and 0.697 for 5 years in the validation set. The calibration curves revealed satisfactory consistency between the prediction of deviation correction and ideal reference line.

**Conclusions:**

The prognostic nomogram developed in the analytical data of SEER it provided high accuracy and reliability in predicting the survival outcomes of primary bladder SRCC patients and could be used to comprehensively assess the risk of SRCC. Moreover, they could enable clinicians to make more precise treatment decisions for primary bladder SRCC patients.

## Introduction

Bladder cancer (BCa) is the second most diagnosed malignant carcinoma of the genitourinary tract, being the 10th tumor for incidence when considering both sexes, and the 7th considering only the men population [1]. The most common type of BCa is urothelial carcinoma, which occurs in 90% of all cases. Most BCas can be diagnosed at an early stage. Although 75% are non-muscle-invasive tumors at first diagnosis, approximately 78% of patients relapse within 5 years. Patients with the same pathologic type of BCa and clinical stage could have different prognoses.

Signet ring cell carcinoma (SRCC), classified as subtypes of adenocarcinoma, most commonly occurs in the stomach and colon. Primary bladder SRCC is a rare type of bladder cancer, accounting for approximately <1% of cases, with a high mortality rate [2, 3]. Because of its rarity, it is impossible to fully comprehensively its clinicopathological features, prognosis and histogenesis [4, 5].

In this regard, although numerous steps in the past have been taken in the management of this pathology, still little knowledge we have of the characteristics that influence its prognosis. Continuing to investigate them is the first step to do to develop new strategies for a more efficacy BCa treatment and management.

A standardized treatment for primary bladder SRCC has not been established and surgery is generally considered the most predominant treatment method, but the optimal surgical approach and whether radiotherapy or chemotherapy cannot be determined by consensus. Primary bladder SRCC may often overlooked by patients and clinicians as its low frequency.

TNM staging system is used by the American Joint Committee on Cancer (AJCC) to predict prognosis and guide treatment in BCa; however, it is not specially designed for SRCC. Ferro et al. [6] found that neutrophil percentage-to-albumin ratio could predict both overall survival (OS) and cancer-specific survival (CSS). They indicated that patients, treated with neoadjuvant chemotherapy and radical cystectomy, with high neutrophil percentage-to-albumin ratio had a longer OS and CSS than low neutrophil percentage-to-albumin ratio. However, many individualized characteristics, such as histologic grade, tumor size, lymph node metastasis, surgery, lymph nodes removed, chemotherapy and radiotherapy, that may be predictive are not involved [5, 7]. Nomograms can present simple statistical analysis and visualization results, which can help physicians make better clinical decisions and to promote personalized medical therapy. Unfortunately, there is no consensus prognostic model for primary bladder SRCC.

Prognostic nomograms are currently used widely in oncologic medicine as prognostic devices. Because the knowledge of the prognosis of primary bladder SRCC is essential for pretherapeutic assessment the aim of the current study was to describe the frequency of occurrence based on the SEER database. Another aim of the study was to construct a nomogram to predict OS in de novo diagnosed primary bladder SRCC patients. The study may also help to choose suitable management strategies by increasing the understanding of prognosis in newly diagnosed primary bladder SRCC patients.

## Methods

### Patients and variables

Around 28% of the US population has cancer incidence in the SEER database that includes 18 cancer registries. The SEER * Stat software (version 8.4.0.1) was used to identify all primary SRCC of the bladder cases reported in the SEER database from 2004 to 2015. We collected the following data: (1) primary bladder SRCC patients; (2) the diagnosis of SRCC was based on the ICD-O-3; coded as 8490/3; (3) confirmed by positive histology and diagnosed by first or only cancer. Those who did not have a histological diagnosis or lacked survival information were excluded.

Patient demographics analyzed included sex, age, race, marital status, and year of diagnosis. Tumor characteristics studied included tumor size, primary site, AJCC T stage, N stage, M stage, and histologic grade. Information on distant metastasis sites only became available after 2010, so the metastasis information was included from 2010 to 2015. Treatment characteristics included surgery, lymph nodes removed, radiation, chemotherapy, and the sequence of surgery between radiation. OS was the main endpoint. A patient‘s OS is defined as the period from the time of first diagnosis to his death or last follow-up.

### Statistical analyses

In the ratio of 1:1, all included samples were randomly split into the training and validation sets. The categorical data were presented as frequency (percentage), and the chi-square test was used to compare groups.

Based on multivariate Cox proportional hazard analysis, we constructed nomograms using the factors with *P* < 0.05. Multivariate analysis using the Cox proportional hazards model enabled the identification of baseline and clinical variables associated with OS time. The Kaplan–Meier survival curves were plotted and compared by log-rank test.

Last but not least, we considered variables with a *P* value of less than 0.05 as independent predictors in the multivariate Cox model and incorporated them into the nomogram model. In addition, both the training and validation sets were validated using the consistency index (C-index), receiver operating characteristic (ROC) curve, and calibration curve. And also to calibrate the prediction capacity of the nomogram for 1-, 3- and 5-year OS. Those who had a C-index and an area under the curve (AUC) of > 0.65 were considered as having an acceptable fit. R software (www.rproject.org) and SPSS software program (version 25.0) were used for statistical analysis. Statistical significance was determined by *P* values < 0.05 (*P* < 0.05).

The study was conducted in accordance with the Helsinki Declaration (revised in 2013). Because cancer is a reportable disease in every state of the US, it is not required that informed patient consent be obtained for the release of data from the SEER database.

## Results

### Baseline characteristics of the overall, training and validation sets

Table 1 presents the demographic and clinicopathological characteristics of 219 patients.

**Table 1:** Baseline demographic and clinicopathologic characteristics for primary bladder SRCC (*n*, %)

Variable	Overall set	Training set	Validation set
Age, years			
< 60	79(36.1%)	40(36.0%)	39(36.1%)
≥ 60	140(63.9%)	71(64.0%)	69(63.9%)
Race			
White	180(82.2%)	92(82.9%)	88(81.5%)
Black	26(11.9%)	14(12.6%)	12(11.1%)
Others	12(5.5%)	4(3.6%)	8(7.4%)
Unknown	1(0.5%)	1(0.9%)	0(0.0%)
Sex			
Female	60(27.4%)	32(28.8%)	28(25.9%)
Male	159(72.6%)	79(71.2%)	80(74.1%)
Marital status			
Single	37(16.9%)	18(16.2%)	19(17.6%)
Married	129(58.9%)	68(61.3%)	61(56.5%)
SDW	43(19.6%)	20(18.0%)	23(21.3%)
Unknown	10(4.6%)	5(4.5%)	5(4.6%)
Year of diagnosis			
2004–2009	113(51.6%)	54(48.6%)	59(54.6%)
2010–2015	106(48.4%)	57(51.4%)	49(45.4%)
Tumor size, cm			
< 4.0	45(20.5%)	27(24.3%)	18(16.7%)
≥ 4.0	60(27.4%)	27(24.3%)	33(30.6%)
Unknown	114(52.1%)	57(51.4%)	57(52.8%)
Primary site			
Trigone of bladder	16(7.3%)	9(8.1%)	7(6.5%)
Dome of bladder	18(8.2%)	10(9.0%)	8(7.4%)
Lateral wall of bladder	26(11.9%)	12(10.8%)	14(13.0%)
Anterior wall of bladder	5(2.3%)	2(1.8%)	3(2.8%)
Posterior wall of bladder	13(5.9%)	7(6.3%)	6(5.6%)
Bladder neck	5(2.3%)	3(2.7%)	2(1.9%)
Ureteric orifice	2(0.9%)	2(1.8%)	0(0.0%)
Urachus	8(3.7%)	5(4.5%)	3(2.8%)
Overlapping lesion of bladder	32(14.6%)	19(17.1%)	13(12.0%)
Bladder, NOS	94(42.9%)	42(37.8%)	52(48.1%)
T Stage			
T1	28(12.8%)	15(13.5%)	13(12.0%)
T2	50(22.8%)	31(27.9%)	19(17.6%)
T3	42(19.2%)	21(18.9%)	21(19.4%)
T4	81(37.0%)	38(34.2%)	43(39.8%)
Tis	1(0.5%)	0(0.0%)	1(0.9%)
TX	17(7.8%)	6(5.4%)	11(10.2%)
N stage			
N0	127(58.0%)	72(64.9%)	55(50.9%)
N1	33(15.1%)	14(12.6%)	19(17.6%)
N2	42(19.2%)	20(18.0%)	22(20.4%)
N3	2(0.9%)	1(0.9%)	1(0.9%)
NX	15(6.8%)	4(3.6%)	11(10.2%)
M stage			
M0	160(73.1%)	82(73.9%)	78(72.2%)
M1	52(23.7%)	24(21.6%)	28(25.9%)
MX	7(3.2%)	5(4.5%)	2(1.9%)
Grade			
II	4(1.8%)	2(1.8%)	2(1.9%)
III	120(54.8%)	52(46.8%)	68(63.0%)
IV	52(23.7%)	35(31.5%)	17(15.7%)
Unknown	43(19.6%)	22(19.8%)	21(19.4%)
Bone metastasis			
No	101(96.2%)	55(98.2%)	46(93.9%)
Yes	4(3.8%)	1(1.8%)	3(6.1%)
Brain metastasis			
No	103(98.1%)	55(98.2%)	48(98.0%)
Yes	2(1.9%)	1(1.8%)	1(2.0%)
Liver metastasis			
No	103(98.1%)	55(98.2%)	48(98.0%)
Yes	2(1.9%)	1(1.8%)	1(2.0%)
Lung metastasis			
No	99(94.3%)	52(92.9%)	47(95.9%)
Yes	6(5.7%)	4(7.1%)	2(4.1%)
Surgery			
None	31(14.2%)	13(11.7%)	18(16.7%)
TURB	62(28.3%)	35(31.5%)	27(25.0%)
Complete cystectomy + reconstruction	49(22.4%)	24(21.6%)	25(23.1%)
Pelvic exenteration	42(19.2%)	23(20.7%)	19(17.6%)
Others	35(16.0%)	16(14.4%)	19(17.6%)
Lymph nodes removed			
None	121(55.3%)	61(55.0%)	60(55.6%)
1 to 3 regional	8(3.7%)	7(6.3%)	1(0.9%)
4 or more regional	82(37.4%)	39(35.1%)	43(39.8%)
Unknown	8(3.7%)	4(3.6%)	4(3.7%)
Radiation			
None/Unknown	172(78.5%)	94(84.7%)	78(72.2%)
Yes	47(21.5%)	17(15.3%)	30(27.8%)
Chemotherapy			
None/Unknown	119(54.3%)	65(58.6%)	54(50.0%)
Yes	100(45.7%)	46(41.4%)	54(50.0%)
Surgery/Radiation sequence			
No radiation and/or surgery	180(82.2%)	98(88.3%)	82(75.9%)
Radiation after surgery	36(16.4%)	12(10.8%)	24(22.2%)
Radiation before and after surgery	3(1.4%)	1(0.9%)	2(1.9%)
Vital status			
Alive	27(12.3%)	14(12.6%)	13(12.0%)
Dead	192(87.7%)	97(87.4%)	95(88.0%)
Survival month	13(6,39)	14(6,41)	12(5.25, 35.75)

*SDW* Divorced + Separated + Widowed + Unmarried or Domestic Partner, *TURB* transurethral resection of the bladder

### Prognostic factor analysis

**Fig. 1:**
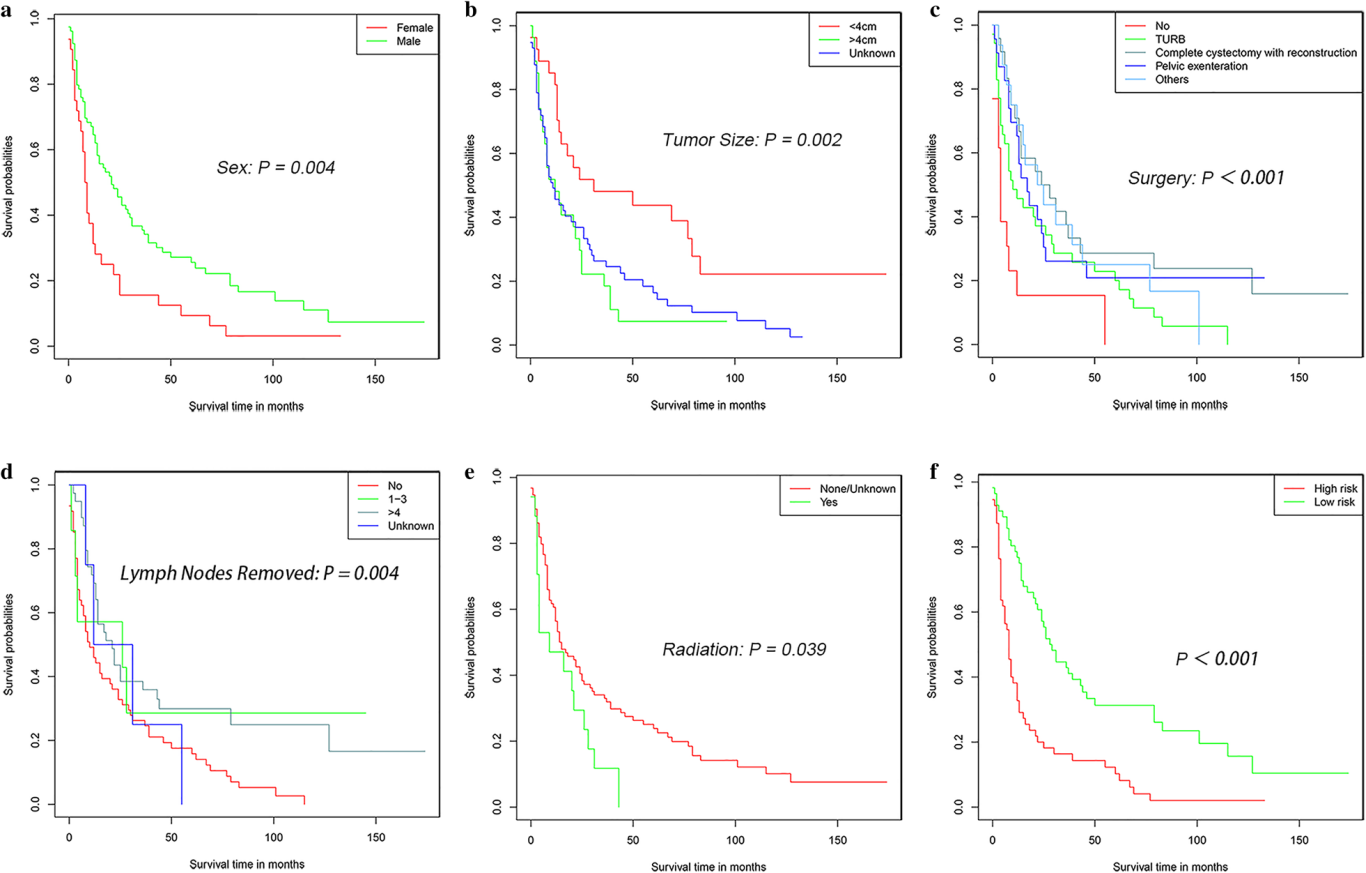
Kaplan–Meier survival curves for OS by subgroups of sex **a**, tumor size **b**, surgery **c**, lymph nodes removed **d**, radiation **e**, and risk level **f**

In this study, univariate analyses confirmed that risk factors of bladder SRCC include sex, race, marital status, tumor size, T stage, N stage, M stage, surgery, lymph node removed, and radiation. However, multivariate analyses showed independent risk factors only include race (Unknown vs. White, HR = 59.085, 95% CI = 2.638 ~ 913.422, *P* = 0.009), marital status(SDW vs. Single, HR = 2.878, 95% CI = 1.296 ~ 6.391, *P* = 0.009), T stage (T2 vs. T1, HR = 2.710, 95% CI = 1.127 ~ 6.518, *P* = 0.026; T3 vs. T1, HR = 6.124, 95% CI = 1.881 ~ 19.924, *P* = 0.003; and T4 vs. T1, HR = 4.141, 95% CI = 1.534 ~ 11.252, *P* = 0.005), N stage (N2 vs. N0, HR = 4.461, 95% CI = 2.016 ~ 9.873, *P* < 0.001), M stage (M1 vs. M0, HR = 2.400, 95% CI = 1.156 ~ 4.983, *P* = 0.019), surgery (Pelvic exenteration vs. None, HR = 0.223, 95% CI = 0.069 ~ 0.726, *P* < 0.012; and Others vs. None, HR = 0.194, 95% CI = 0.064 ~ 0.591, *P* = 0.004), and lymph node removed (4 or more regional vs. None, HR = 0.299, 95% CI = 0.127 ~ 0.703, *P* = 0.006; and Unknown vs. None, HR = 0.120, 95% CI = 0.023 ~ 0.627, *P* = 0.012) (Table 2). The Kaplan–Meier survival curves shown in Fig. 1 show the OS of the training set patient population by subgroups of sex, tumor size, surgery, lymph nodes removed, and radiation (Fig. 1a–e). The median OS was only 14 months. The median OS, respectively, were 4, 10, 24, 17, and 22 months for none, transurethral resection of the bladder (TURB), complete cystectomy with reconstruction, pelvic exenteration and others surgery (*P* < 0.001). The none lymph nodes removed, 1 to 3 regional, 4 or more regional, and unknown were 10, 26, 21 and 12 months, respectively (*P* = 0.004). Figure 1f shows that, compared with high-risk patients, low-risk patients tended to have a better OS (*P* < 0.001).

**Table 2:** Univariate and multivariable analyses of prognostic factors for primary bladder SRCC

Variables	Univariate analysis			Multivariate analysis		
	HR	95% CI	*P* value	HR	95% CI	*P* value
Sex						
Female	Reference			Reference		
Male	0.536	0.348 ~ 0.825	0.005^a^	0.586	0.329 ~ 1.041	0.068
Age, year						
< 60	Reference					
≥ 60	1.138	0.749 ~ 1.728	0.545			
Race						
White	Reference			Reference		
Black	1.292	0.717 ~ 2.327	0.394	1.775	0.839 ~ 3.753	0.133
Others	2.157	0.781 ~ 5.956	0.138	2.265	0.558 ~ 9.183	0.252
Unknown	66.305	6.556 ~ 670.578	< 0.001^b^	59.085	2.638 ~ 913.422	0.009^a^
Marital status						
Single	Reference			Reference		
Married	0.837	0.472 ~ 1.478	0.540	1.264	0.629 ~ 2.540	0.511
SDW	3.416	1.709 ~ 6.830	< 0.001^b^	2.878	1.296 ~ 6.391	0.009^a^
Others	0.632	0.209 ~ 1.909	0.415	1.758	0.485 ~ 6.372	0.390
Primary site						
Trigone of bladder	Reference					
Dome of bladder	0.890	0.317 ~ 2.494	0.825			
Lateral wall of bladder	1.346	0.520 ~ 3.487	0.540			
Anterior wall of bladder	1.487	0.308 ~ 7.175	0.621			
Posterior wall of bladder	1.127	0.377 ~ 3.367	0.830			
Bladder neck	1.252	0.322 ~ 4.853	0.745			
Ureteric orifice	0.386	0.046 ~ 3.207	0.378			
Urachus	0.646	0.188 ~ 2.217	0.487			
Overlapping lesion of bladder	1.464	0.603 ~ 3.556	0.400			
Bladder, NOS	1.443	0.643 ~ 3.239	0.374			
Tumor size, cm						
< 4.0	Reference			Reference		
≥ 4.0	2.437	1.326 ~ 4.478	0.004^a^	1.969	0.978 ~ 3.962	0.058
Unknown	2.058	1.215 ~ 3.486	0.007^a^	1.937	0.997 ~ 3.764	0.051
Grade						
II	Reference					
III	3.246	0.446 ~ 23.650	0.245			
IV	2.734	0.372 ~ 20.090	0.323			
Unknown	2.083	0.278 ~ 15.610	0.475			
T stage						
T1	Reference			Reference		
T2	1.555	0.782 ~ 3.093	0.208	2.710	1.127 ~ 6.518	0.026^a^
T3	2.486	1.186 ~ 5.212	0.015^a^	6.124	1.881 ~ 19.924	0.003^a^
T4	2.569	1.307 ~ 5.049	0.006^a^	4.141	1.534 ~ 11.252	0.005^a^
TX	3.263	1.209 ~ 8.805	0.020^a^	1.455	0.394 ~ 5.365	0.574
N stage						
N0	Reference			Reference		
N1	1.688	0.915 ~ 3.115	0.094	2.242	0.900 ~ 5.604	0.085
N2	2.247	1.309 ~ 3.859	0.003^a^	4.461	2.016 ~ 9.873	< 0.001^b^
N3	2.116	0.289 ~ 15.484	0.461	6.957	0.739 ~ 65.460	0.090
NX	2.214	0.780 ~ 6.139	0.127	0.954	0.190 ~ 4.790	0.954
M stage						
M0	Reference			Reference		
M1	3.898	2.355 ~ 6.452	< 0.001^b^	2.400	1.156 ~ 4.983	0.019^a^
MX	1.317	0.528 ~ 3.287	0.554	0.982	0.235 ~ 4.095	0.980
Surgery						
None	Reference			Reference		
TURB	0.498	0.256 ~ 0.972	0.040^a^	0.539	0.203 ~ 1.431	0.215
Complete cystectomy + reconstruction	0.275	0.131 ~ 0.579	< 0.001^b^	0.362	0.116 ~ 1.131	0.080
Pelvic exenteration	0.371	0.178 ~ 0.777	0.008^a^	0.223	0.069 ~ 0.726	0.012^a^
Others	0.349	0.160 ~ 0.762	0.008^a^	0.194	0.064 ~ 0.591	0.004^a^
Lymph nodes removed						
None	Reference			Reference		
1 to 3 regional	0.522	0.207 ~ 1.315	0.168	0.367	0.090 ~ 1.498	0.163
4 or more regional	0.540	0.345 ~ 0.867	0.007^a^	0.299	0.127 ~ 0.703	0.006^a^
Unknown	0.852	0.309 ~ 2.350	0.756	0.120	0.023 ~ 0.627	0.012^a^
Surgery/Radiation sequence						
No radiation and/or surgery	Reference					
Radiation after surgery	1.773	0.958 ~ 3.283	0.068			
Radiation before and after surgery	1.046	1.145 ~ 7.554	0.965			
Radiation						
None/Unknown	Reference			Reference		
Yes	1.773	1.024 ~ 3.070	0.041^a^	0.552	0.227 ~ 1.343	0.190
Chemotherapy						
None/Unknown	Reference					
Yes	1.078	0.713 ~ 1.630	0.722			

*SDW* Divorced + Separated + Widowed + Unmarried or Domestic Partner, *TURB* transurethral resection of the bladder

^a^*P* < 0.05

^b^*P* < 0.001

### Development and validations of predicting nomograms for OS

**Fig. 2:**
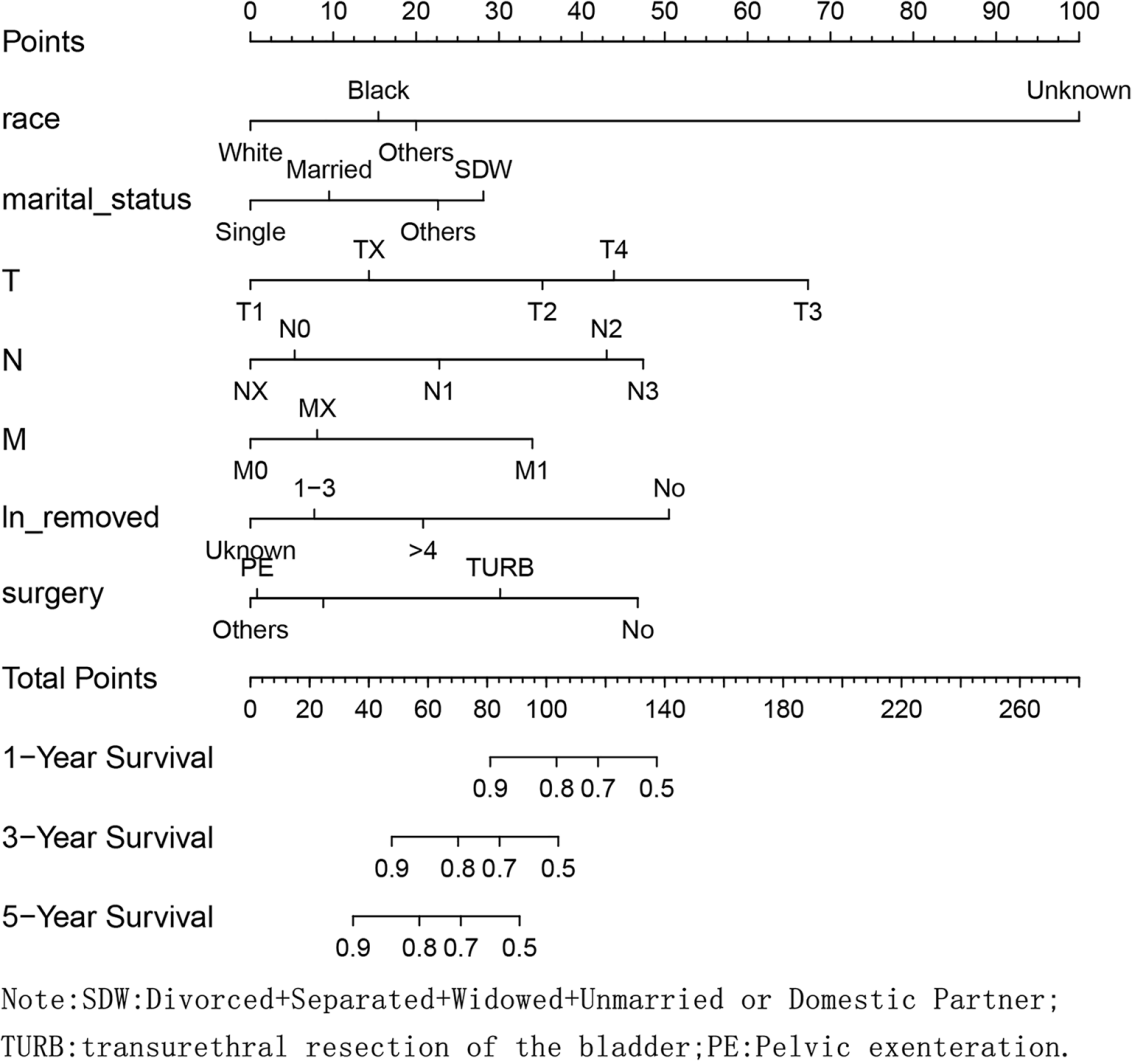
Prognostic nomogram predicting 1-, 3- and 5-year overall survival rate in primary bladder SRCC patients

**Fig. 3:**
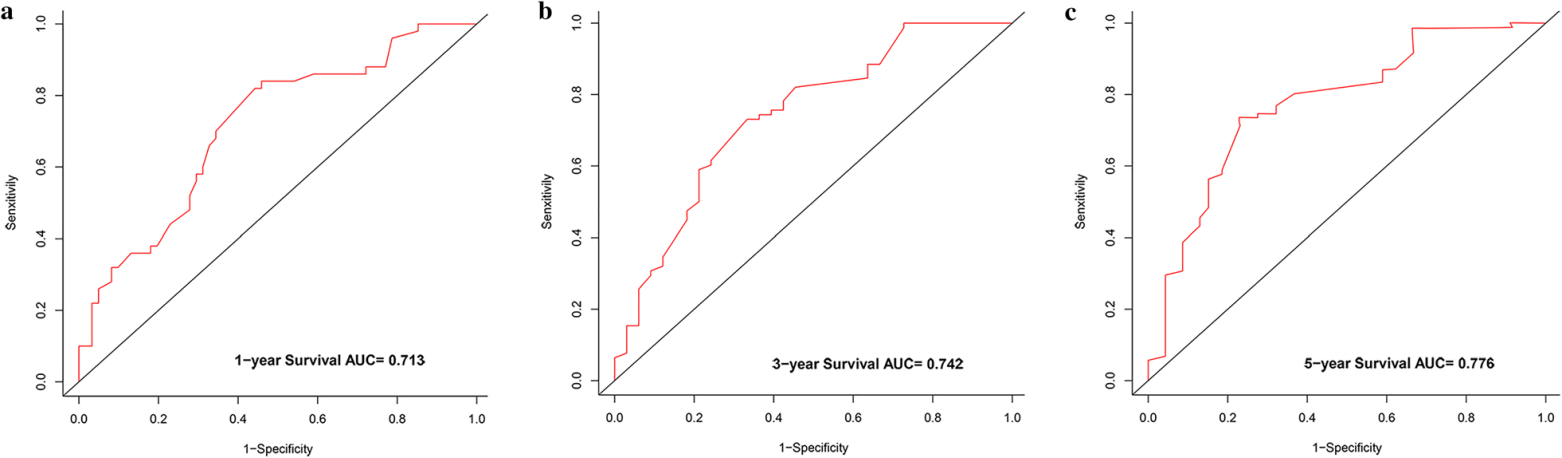
ROC curve for predicting patient survival at **a** 1, **b** 3, and **c** 5 years in the training set

**Fig. 4:**
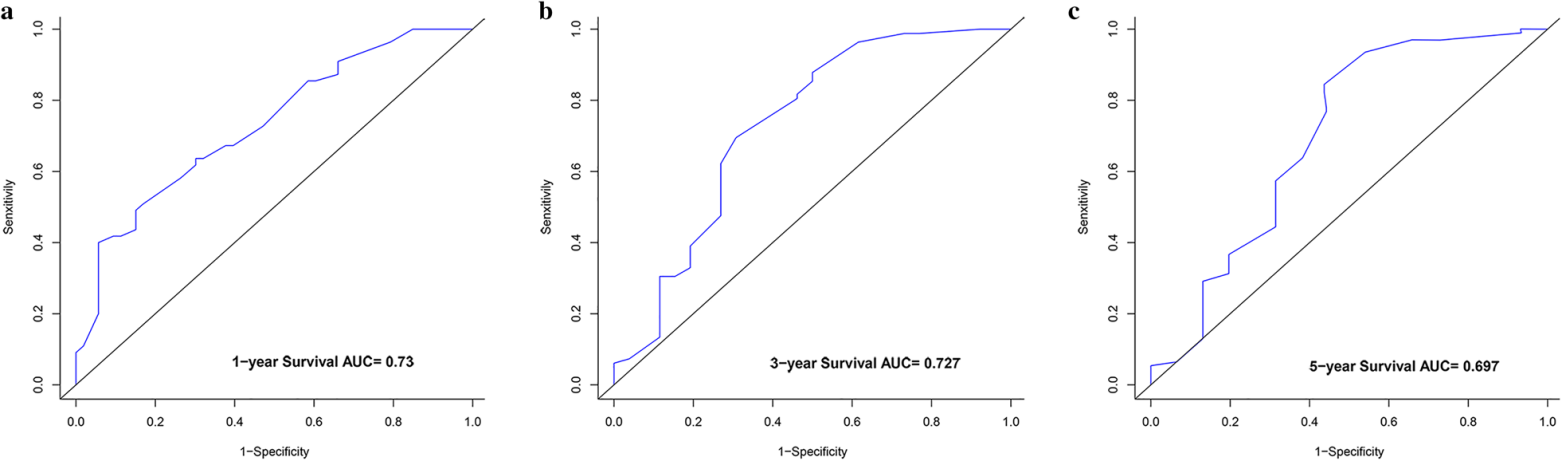
ROC curve for predicting patient survival at **a** 1, **b** 3, and **c** 5 years in the validation set

Based on the significant risk factors, the nomogram for predicting 1-, 2- and 3-year OS was developed using Cox multivariate analysis (Fig. 2). The C-index and ROC curves were compared to determine, whether the survival months predicted by the nomograms were in accordance with the actual survival times. The C-index of the nomogram OS was 0.771 and 0.715 in the training and validation sets, respectively. The ROC curve estimation of the nomogram in the training set also showed acceptable accuracy, with a 1-, 3-, and 5-year AUC of 0.713, 0.742 and 0.776, respectively (Fig. 3a–c). In addition, the validation set were 0.730, 0.727 and 0.697, respectively (Fig. 4a–c). These results indicated that the model we constructed was relatively accurate.

**Fig. 5:**
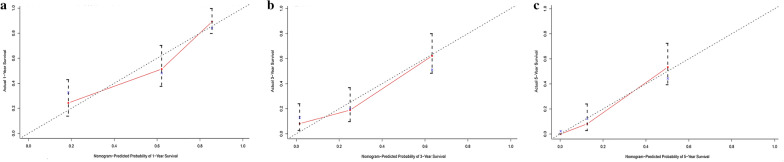
Calibration curve for predicting patient survival at **a** 1, **b** 3, and **c** 5 years in the training set

**Fig. 6:**
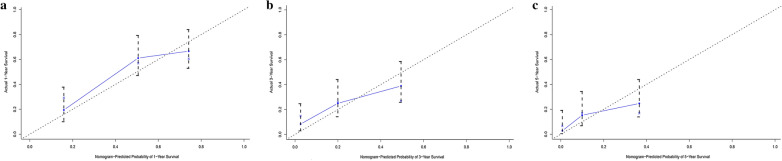
Calibration curve for predicting patient survival at **a** 1, **b** 3, and **c** 5 years in the validation set

More importantly, we calibrated the 1-, 3-, and 5-year OS nomogram in both the training and validation sets. The calibration plots showed that the nomogram had a favorable predictive accuracy in both the training set (Fig. 5a–c) and validation set (Fig. 6a–c). This result indicated good agreement between the nomogram predictions and the observed results in the training and validation sets.

## Discussion

The primary bladder SRCC is a rare form of bladder cancer, with an incidence rate of less than 0.6% [8, 9]. It was less commonly reported, since the first patient was reported by Saphir [10]. Due to the rarity of bladder SRCC, most reports were case reports or reviews of previous cases [4, 11, 12].

The present study showed that lymph node metastasis did not occur in 58.0% overall patients and distant metastasis did not occur in 73.1% overall patients. The bladder SRCC had a lower rate of lymph node metastases and distant metastases, but their prognoses were worse. As previously described, SRCC presents a higher histological grade rate and advanced stage disease, which could induce a poor prognosis [5, 13, 14]. According to the literature reported, the survival rate of bladder SRCC was lower than urothelial carcinoma, and the natural duration of the disease was 3.5 months [4]. A Japanese study, reported by Dadhania et al. [15] also demonstrated that almost half of the bladder SRCC patients were already at AJCC stage IV at the time of diagnosis, and the median survival time was approximately 8 months. Besides, the surviving period of time did not exceed 2 years. In the analytical data of SEER it showed, the median survival was 13, 14, and 12 months, respectively, for oveall, training and validation sets.

As for the prognostic factors of bladder SRCC, clinical study results were inconsistent.

Multiple studies, compared with bladder SRCC and urothelial carcinoma, have shown that histological type, cystectomy, marital status, histological grade, year of diagnosis and sex have been identified as prognostic factors. In addition, SRCC, histological type, is an independent risk factor for Bca. [4, 14]. Wang et al. [13] suggested that patient characteristics such as age, marital status, AJCC stage, and cystectomy were independent predictors of survival for bladder SRCC patients. In this study, univariate analyses confirmed that risk factors of bladder SRCC include sex (Male vs. Female, HR = 0.536, 95% CI = 0.348 ~ 0.825, *P* = 0.005), race (Unknown vs. White, HR = 66.305, 95% CI = 6.556 ~ 670.578, *P* < 0.001), marital status (SDW vs. Single, HR = 3.416, 95% CI = 1.709 ~ 6.830, *P* < 0.001), tumor size (≥ 4.0 cm vs. < 4.0 cm, HR = 2.437, 95% CI = 1.326 ~ 4.478, *P* = 0.004; and Unknown vs. < 4.0 cm, HR = 2.058, 95% CI = 1.215 ~ 3.486, *P* = 0.007), T stage (T3 vs. T1, HR = 2.486, 95% CI = 1.186 ~ 5.212, *P* = 0.015; T4 vs. T1, HR = 2.569, 95% CI = 1.307 ~ 5.049, *P* = 0.006; and TX vs. T1, HR = 3.263, 95% CI = 1.209 ~ 8.805, *P* = 0.020), N stage (N2 vs. N0, HR = 2.247, 95% CI = 1.309 ~ 3.859, *P* = 0.003), M stage (M1 vs. M0, HR = 3.898, 95% CI = 2.355 ~ 6.452, *P* < 0.001), surgery (TURB vs. None, HR = 0.498, 95% CI = 0.256 ~ 0.972, *P* = 0.040; Complete cystectomy + reconstruction vs. None, HR = 0.275, 95% CI = 0.131 ~ 0.579, *P* < 0.001; Pelvic exenteration vs. None, HR = 0.371, 95% CI = 0.178 ~ 0.777, *P* = 0.008; and Others vs. None, HR = 0.349, 95% CI = 0.160 ~ 0.762, *P* = 0.008), lymph nodes removed (4 or more regional vs. None, HR = 0.540, 95% CI = 0.345 ~ 0.867, *P* = 0.007), and radiation (Yes vs. None/Unknown, HR = 1.773, 95% CI = 1.024 ~ 3.070, *P* = 0.041). However, multivariate analyses showed independent risk factors only include race (Unknown vs. White, HR = 59.085, 95% CI = 2.638 ~ 913.422, *P* = 0.009), marital status(SDW vs. Single, HR = 2.878, 95% CI = 1.296 ~ 6.391, *P* = 0.009), T stage (T2 vs. T1, HR = 2.710, 95% CI = 1.127 ~ 6.518, *P* = 0.026; T3 vs. T1, HR = 6.124, 95% CI = 1.881 ~ 19.924, *P* = 0.003; and T4 vs. T1, HR = 4.141, 95% CI = 1.534 ~ 11.252, *P* = 0.005), N stage (N2 vs. N0, HR = 4.461, 95% CI = 2.016 ~ 9.873, *P* < 0.001), M stage (M1 vs. M0, HR = 2.400, 95% CI = 1.156 ~ 4.983, *P* = 0.019), surgery (Pelvic exenteration vs. None, HR = 0.223, 95% CI = 0.069 ~ 0.726, *P* < 0.012; and Others vs. None, HR = 0.194, 95% CI = 0.064 ~ 0.591, *P* = 0.004), and lymph node removed (4 or more regional vs. None, HR = 0.299, 95% CI = 0.127 ~ 0.703, *P* = 0.006; and Unknown vs. None, HR = 0.120, 95% CI = 0.023 ~ 0.627, *P* = 0.012) (Table 2). Data from our study and a previous studies proved that marital status and surgery were independent risk factors for bladder SRCC. However, our study did not show age, sex, and histological grade et. are prognostic factors.

Our study suggests that race, marital status, T stage, N stage, M stage, surgery, and lymph node removed were independent prognostic factors for OS in bladder SRCC patients. Using the nomogram we developed and validated, we were able to predict the 1-, 3-, and 5-year OS for bladder SRCC patients with good discrimination and high accuracy. Notably, radiation and chemotherapy were not significantly associated with the OS and were not present in the nomograms.

However, due to the rarity of the bladder SRCC and the lack of randomized clinical trials, there are no accepted guidelines for treating it. The most common surgery for bladder SRCC was TURB (62, 28.3%), and followed by radical cystectomy with reconstruction (49, 22.4%) in overall set. Due to infiltrative pattern of growth and early propensity of metastasis, nevertheless, certain scholars [16, 17] reported that TURB and partial cystectomy carry the risk of tumor recurrence. Therefore, radical cystectomy appears to be the treatment of first choice [18–20]. A recent systematic review concluded that all muscle invasive BCa patients, which accompanied with histological variants, including SRCC, should perform radical cystectomy [7]. Meanwhile, Guo et al. found that radical cystectomy with lymphadenectomy was associated with improved OS in SRCC patients when compared with radical cystectomy alone [21]. Recently, 70% patients were reportedly treated with surgery [22]. The number of patients with surgery (188, 85.8%) in the overall set was higher than the above values, suggesting that clinicians already have more knowledge of this disease and have attracted increasing attention for surgery. Some Scholars have suggested that bladder SRCC patients would benefit from adjuvant chemotherapy [23, 24]. However, our result shows that bladder SRCC can not benefit from postoperative adjuvant chemotherapy. The neoadjuvant nature of chemotherapy has some benefits: delivering effective systemic therapy for micrometastases; improvement of direct drug delivery into the bladder and to surrounding lymphatic vessels and lymph nodes; and improves the performance status of naive patients undergoing major surgery [25]. It can be downgrade of disease and an overall improved prognosis. However, a high-grade toxicity in more than one patient out of three, without a significant OS improvement in this patients. Therefore, the selection of patients that could benefit from neoadjuvant chemotherapy avoiding toxicity is a challenge for the future [26]. Recently, immunity checkpoint inhibitors have been introduced as an alternative treatment option for bladder SRCC patients [27]. The molecular biology represents the key to reveal the possible Achilles’ heel in BC as well as identifying novel targets. A consensus, identified 6 consensus muscle invasive bladder cancer: Luminal-papillary (LumP), Luminal-Unstable (LumU), Luminal-non-Specified (LumNS), Basal/Squamous (Ba/Sq), Stromarich, and Neuroendocrine-like (NE-like), was published in 2020 in the European Urology Journal [28]. In several phase II trials, immunotherapy was shown to be a valuable strategy for neoadjuvant therapy, especially for patients who were unable to receive cisplatin [29].

In summary, further multicenter clinical trial should be conducted to treatment bladder SRCC. Most importantly, our clinical prognostic model was innovative, rational and clinically feasible, which could be an accessible prognosis tool in clinical practice.

Several limitations are noteworthy. The first was that it was retrospective. In addition, there is little detailed information on chemotherapy drugs. Finally, The predictive accuracy of the nomogram for OS needed to be validated by external cohorts.

## Conclusions

Our study shows that race, marital status, T stage, N stage, M stage, surgery and lymph node removed were independent predictive factors of OS in bladder SRCC patients. We established a nomogram based on the above parametric, and identified the high accuracy and reliability in estimating the survival of individual patients. Thus, the nomogram could be an accessible prognosis tool in clinical practice and provide personalized treatment plans.

## Data Availability

The data sets generated and/or analyzed during the current study are available in the SEER repository, https://seer.cancer.gov/.

## Abbreviations

SRCC: Signet ring cell carcinoma
C-index: Consistency index
AUC: Area under the curve
OS: Overall survival
CSS: Cancer-specific survival
BCa: Bladder cancer
US: United States
ROC: Receiver operating characteristic
TRUB: Transurethral resection of the bladder
SEER: Surveillance, epidemiology, and end results
AJCC: The American joint committee on cancer
HR: Hazard ratio
CI: Confdence interval
SDW: Divorced + Separated + Widowed + Unmarried or Domestic Partner
PE: Pelvic exenteration
